# Community stakeholders’ views on reducing violence against women in Pakistan

**DOI:** 10.1186/s12905-020-00961-3

**Published:** 2020-05-07

**Authors:** Tazeen Saeed Ali, Rozina Karmaliani, Hussain Maqbool Ahmed Khuwaja, Nasim Zahid Shah, Zahid Hyder Wadani, Saher Aijaz, Asli Kulane

**Affiliations:** 1 School of Nursing and Midwifery, Aga Khan University (SONAM AKU), Stadium Road, Karachi, Sindh Pakistan; 2 Umeed-e-Nau Innovations Center of Excellence in Women and Child Health (COE), Aga Khan University (AKU), Karachi, Pakistan; 3 Department of Equity and Policy Development at Karolinska Institutet, Stockholm, Sweden

**Keywords:** Violence against women, community’s perception of violence, Violence reduction, Interventions to reduce violence

## Abstract

**Background:**

Nearly half of the women experience violence across their lifespan in all the provinces of Pakistan at an alarming rate. Despite knowing the prevalence, there has been meager progress in developing strategies to combat violence at individual, family, or community level. Many interventions suggested in other countries have been pilot tested but the effects of those interventions had been limited. Therefore, the aim of this study is to understand the voices of stakeholders to reduce Violence Against Women (VAW) and to explore the possible community-based strategies that could be implemented in Pakistan.

**Methods:**

A total of 14 Key Informant Interviews (KIIs) and 18 Focus Group Discussions (FGDs) were held across all four provinces of Pakistan. Participants were purposefully recruited and all the interviews were audio-recorded. Transcriptions were open coded and content analysis was done to emerge codes, categories and themes. Ethical approval was obtained from Aga Khan University Ethics Review Committee.

**Results:**

Three major themes emerged on community members and stakeholders’ views on VAW: a) community’s perception of VAW b) the repercussions of VAW, and c) multiple voices regarding strategies to reduce VAW. Participants voiced the need of standing against the status quo, role of awareness and education: regarding capacity building skills, promotion of women rights and women empowerment through Life Skills Based Education (LSBE) through national health works program, has been proposed as an innovative strategy to reduce VAW.

**Conclusions:**

The responsibility to bring about a substantial change in behavior and attitudes must begin with engaging men in all the interventions that aim to reduce violence. Since, VAW is very much linked with the cultural norms, so, without community stakeholder’s involvement and participation it could never be reduced. Keeping the existing socio-cultural dynamics in mind, the need of time is to design and implement innovative interventions that are culturally and contextually appropriate and can be expanded across the country.

## Background

VAW is a prevailing social and public health concern calling for special attention for the mitigation of several physical, psychosocial and emotional health challenges faced by women around the world. VAW encompasses physical, sexual and intimate partner violence leading to increase in Disability Adjusted Life Years (DALYs) and reduced Quality Adjusted Life Years (QALYs) amongst women [1]. Globally, an estimated 4–49% of females between the ages 14–71 years reported of being subjected to physical and other forms of violence from their spouse or intimate partners [2, 3]. In Pakistan, 70–90% of the married women experiences any form of violence from their intimate partners [4, 5].

VAW is largely attributable to socio-cultural norms and family dynamics across various geographic locations [6]. In the developing world, patriarchal mindsets and perceived societal values are amongst the significant factors leading to low status of females in the society. In the context of Pakistan, gender roles are constructed around the combination of traditional roots and societal values, primarily based on the concepts of production and reproduction, taken to mirror masculine and feminine traits of an individual [7]. The women in Pakistan are generally expected to rear children, perform household chores, be submissive, and obey their husbands and families. It has been observed as a common practice for husbands and in-laws to restrict women’s autonomy and limit their rights and participation in decision-making, consequently, increasing the likelihood of women and girls becoming the victim of violence [5, 8]. Domestic violence is considered as a private matter, leading to normalization, denial and resilience among the victims in these areas which further entails inadequacy in reporting and documentation of such incidents in Pakistan [9].

Elimination of all forms of VAW is inherent for Sustainable Development Goals (SDGs), as several programs and policies have been devised and implemented across the globe to address VAW [10]. In Pakistan, the legislature has an extensive framework of legal instruments including Constitutional Provisions and Penal Codes that outlines VAW and also protects the right of women by recognizing it as a punishable offence [11, 12]. The Domestic Violence (Prevention & Protection) Act 2012, protects women and children from physical, psychological and economic harm; however, inadequate implementation of these laws and underreporting of VAW cases has become a significant underline factor for high prevalence of the phenomenon in Pakistan [11, 13]. In addition to legal reforms, several Non-Governmental Organizations (NGOs), government departments and media have been actively engaged in promoting innovative strategies like Life Skills Based Education (LSBE) through messages, campaigns, and activities to increase the awareness for minimizing the act of VAW in community at large by remodeling sociocultural norms and behaviors that normalizes VAW across the country.

**Fig. 1:**
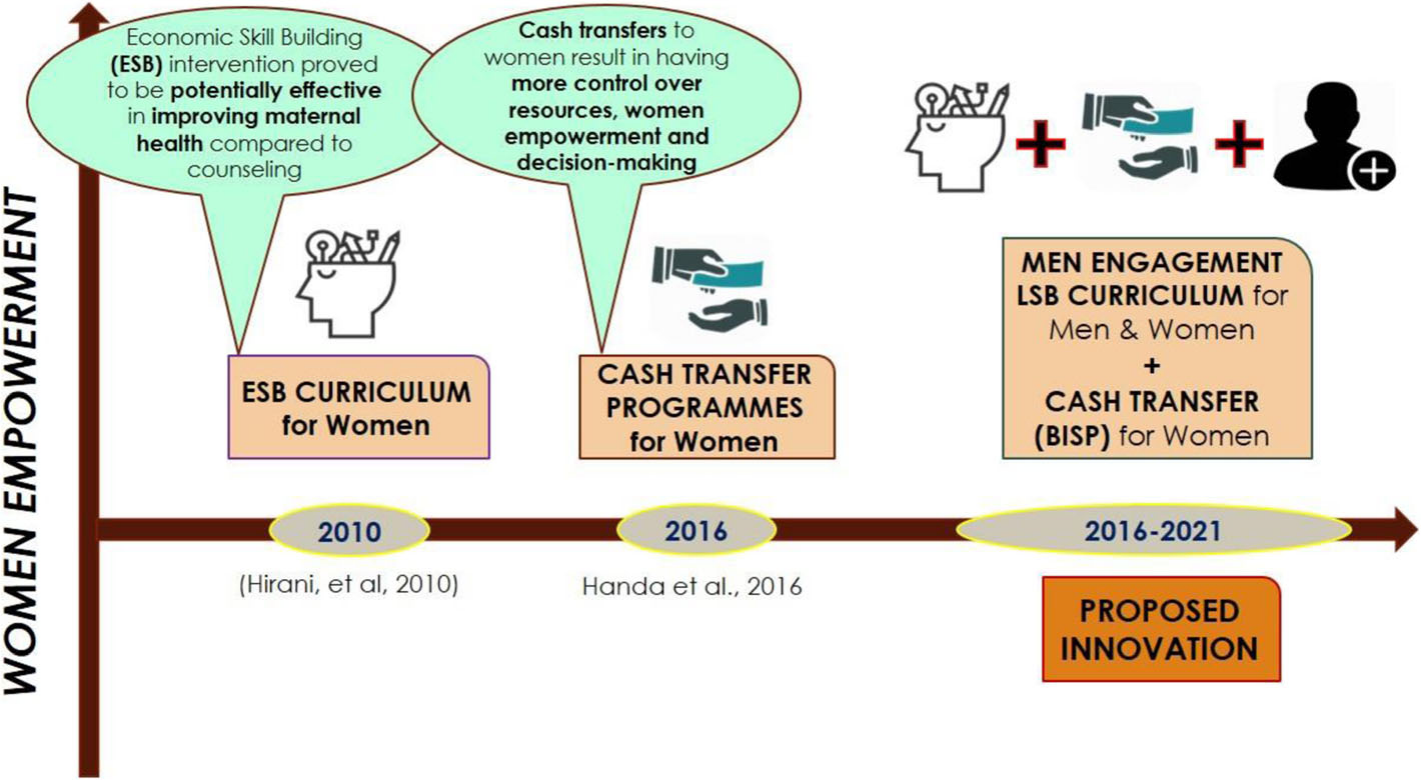
Illustrates the concept development timeline for current intervention involving Men engagement in Women empowerment

To the best of our knowledge, the nationwide implementation and internalization of the innovative strategies has not yet been explored in Pakistan from the perspective of community stakeholders. Therefore, the current research was needed with a rationale to explore the contextual perspectives for gaining access to broad range of information on VAW and to develop the recommendations at national level specifically for identifying strategies to discourage these perceived norms that mainstream VAW (Fig. 1).

## Methods

Using an exploratory-descriptive study design, the research was conducted from August 2016 to February 2018 in the four provinces of Pakistan; Sindh (Thatta), Baluchistan (Lasbela), Punjab (Rahim Yar Khan) and Khyber Pukhtunkhuwa (KPK) (Peshawar). The purpose of conducting the study across four provinces was to record similarities and differences between cultures regarding the phenomenon under study. FGDs and KIIs were conducted to capture the opinions, experiences and personal views from those stakeholders and community members who were residing and well versed with the culture and context of their area.

The integrated socio-ecological model of Heise was adopted as the theoretical frame work for understanding the multifaceted origin of VAW resulting from interplay among individual, situational and socio-cultural factors within the community [14]. At the individual level, the factors highlighted are male gender, substance abuse, and witnessing the abuse of parents as a child. At the relationship level, marital conflict, male control of resources and male decision-making power are of importance. While at the community level, economic realities and unemployment play a role, as does association with antisocial peers and isolation of the family, where unemployment and inability to fulfill male expectations, lack of social support, low socio-economic status increase the risk of violence. Finally, at the societal level, the structures in society influence all other levels. The societal norms and values are of major importance such as women’s status in society, rigid gender roles with a pronounced masculine role and also the level of social acceptability of violence. At this level, gender roles are constructed as are the political, economic and social structures of each society [15, 16].

### Participatory reflection and action (PRA)

Engaging community men and women as the key stakeholders was critical for gaining access to broad range of information regarding VAW. The involvement of key stakeholders will not only provide an opportunity for better contextual understanding, but it will guide towards interpretation of findings and development of recommendations, hence creating a sense of local ownership of results. The approach of community PRA is considered therefore, to best serve the purpose in gathering views of the community. PRA is a systematic approach that enables community men and women to find effective solutions to problems they confront in their everyday lives. PRA is a vehicle for social change through which the participants’ views inform the direction of the project by devising and implementing an action research plan. Evidence suggests PRA a well acknowledged approach addressing community based health related issues and combating gender based violence [17, 18].

This research adopted PRA approach involving meaningful participation of both men and women in formulating a contextually relevant women empowerment intervention to reduce VAW. Qualitative data collection methods were employed in a methodological triangulation framework combining FGDs and KIIs. Use of PRA during qualitative enquiry helped the research team to gain an in-depth understanding of: existing status of the women in the larger picture of family dynamics over her span of life. Additionally, it helped to understand what the act of violence means to a community men and women, couples’ perception of role of men in women empowerment, and factors which will help in contextualizing strategies that can best reduce VAW, keeping the family harmony intact in the long run.

### Participant recruitment and sampling

The participants for FGDs included Benazir Income Support Program (BISP) Cash Transfer (CT) beneficiary couples or couples belonging to similar socio-economic status (SES) from other provinces. For KIIs, BISP Beneficiary Committees (BBCs) members, provincial/district BISP representatives, social mobilizers, community workers and health care professionals were included. There were two separate teams (male & female) comprised of one moderator and one note taker assigned for conducting FGDs with males and females and KIIs. The locally hired team members were well versed with the local context and languages. The moderators were trained by the research team on the content and pedagogy for conducting the interviews. All the interviews were conducted in local languages to maintain the comfort level of the interviewees and to gain an in depth understanding of the issue under discussion.

### Data collection tool

A semi-structured interview guides were developed for conducting FGDs and KIIs. The interview guides were developed by reviewing the available literature, ecological model, and mutual consultation with experts within the team an English version has been uploaded as a supplementary document. The interview guides focused on:

1. Understanding of gender roles in the community: In this part the questions were asked about understanding roles of men and women in the community. It also included perceptions regarding difficulties of growing up as an individual particularly being grown up as a community woman, and positive and negative influences of different relationships across lifespan. Further their experiences and opinions regarding women’s participation in family decision making process, perceived barriers, enablers and role of men in empowering women in the community.
2. The masculinity: In this part questions were asked about experiences and perceptions of community men and women regarding masculinity. It also included definition of a valuable man, attributes of men demonstrating negative masculinity, the barriers and facilitators for attaining valuable manhood in local context and association between positive masculinity and healthy society.
3. The VAW: This part focused on perceptions about VAW, drivers of VAW at household level, awareness of women rights and effects and consequences of violence on family’s health. It also included opinions regarding feasible and innovative interventions at household and community level to overcome VAW and build a healthy environment.

### Data collection

A total of 14 KIIs were conducted. The views of 167 participants (male 88, female 79) were captured by conducting 18 FGDs (9 FGDs with males and 9 with and females) across the study sites. The FGDs were conducted with an average of 6 to 10 participants with a duration of 60 to 90 min per FGD. The FGD participants were homogenous with respect to age (18-70 years), gender, and SES.

**Fig. 2:**
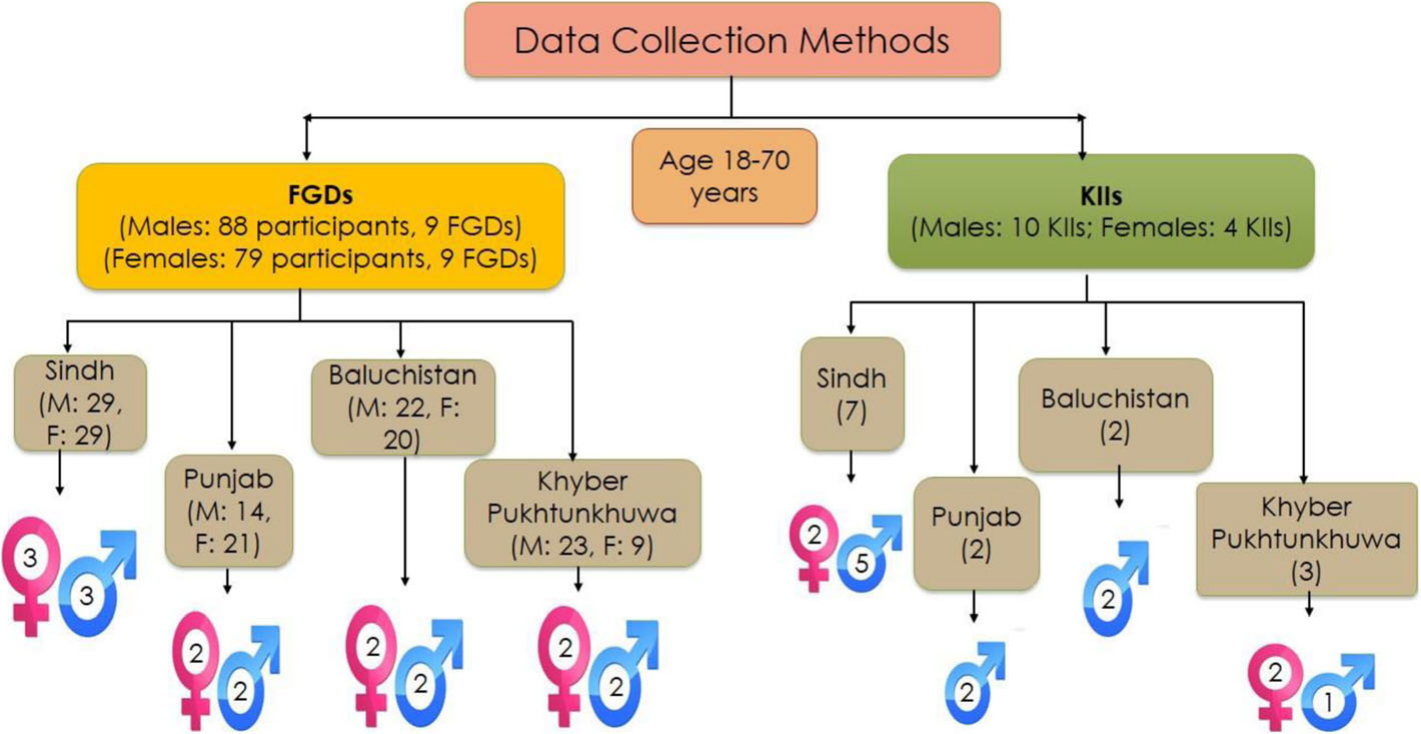
Showing the data collection methods used with age and gender-wise distribution

Each KII lasted for a duration of 30 to 45 min. Details of the KIIs and FGDs are illustrated in Fig. 2. All the interviews were tape recorded after taking informed consent from the participants. In order to maintain confidentiality, the names of the participants were kept anonymous and each transcript was given a unique identifier. The data was kept under lock and key and accessible only to research and audit team.

### Data analysis

All FGDs and KIIs were transcribed and translated into English. The transcripts were read thoroughly and manually coded independently by the research team. Content analysis approach was employed to analyze the data [19]. Researchers began by familiarizing with the data, generating codes, searching, reviewing and naming themes. Relevant categories were picked from the transcribed data and linked to the different themes. All disagreements were discussed and consensus reached on the overall analysis. All the transcripts were reviewed to ensure the necessary information been captured. Credibility was maintained through a repetitive and detailed assessment of data, field notes and regular meetings with the team supervisor. To avoid any fabrication or wrong interpretation of data the conformability was maintained by analysis of data under direct supervisor of the lead researchers. In addition, all the record of collected data was maintained to achieve dependability, and clear, understandable directives were given for data collection and extraction of findings for transferability. This process facilitated the researchers in deciding the level of applicability of results in their settings and maintain trustworthiness of the study.

## Results

The results of the KIIs and FGDs are discussed in line with the three selected domains of the study which assessed perceptions of community with regards to understanding of gender roles in the community, concept of masculinity, understanding and acceptance of acts of violence in the society and strategies to reduce such acts in the society.

### Theme I: Community’s perception of VAW

#### Community’s understanding of VAW

The accounts of the community stakeholders highlighted that physical abuse is not the only form of VAW, it exists in many forms. In the Pakistani context, the most common form of violence is depriving women of their basic rights, property and finance, which is relatively more common in rural areas. Likewise, lack of recognition at household, workplace and community level, hence ignoring and discouraging women, were among the precursors of VAW in Pakistan.

The community men and women describe violence as physical and mental abuse i.e. beating, rape, harassment, bullying, disrespect, suppressing women, depriving women from food and marrying her without consent. According to a participants VAW is explained as:

*“Provision of mental torture, not allowing the women to express her wishes, marrying her without consent, stopping her from getting education”*

[FGD, Peshawar]

#### Underlying factors explaining the act of VAW

The community men and women identified poverty, lack of education, ignorance, unemployment, and substance abuse among the root causes impacting gender roles, violent behaviors and eventually triggering the act of violence in their immediate environments. Moreover, role of technology are other factors contributing, as explained by a participant:

*“Lack of education, substance abuse and unemployment are major reasons for negative masculinity (violent behaviors against women)”*

*[FGD Thatta]*

*“Violence happens because men enforce their choices on females because women are unaware about their rights”*

[FGD Peshawar]

*“Undesired and unwarranted communication such as rapid growth of technology can be thought of as a reason for violence. Secondly, with decline of our religious morals, I think the reconciliatory force between a couples in the form of religious figures like clerics have decreased. People do not wish to speak to local cleric anymore regarding their issues” [KII Thatta]*

#### Sufferers and the wrongdoers

The community men and women shared that women and children are the most vulnerable to be affected by violence. The accounts further highlighted that ill-minded, ignorant and illiterate men perpetrate the act of VAW and children, however, in-laws and village chief (wadhera) were also notified as other perpetrators.

Moreover, Family pressures also promote VAW which is more prevalent in upper Sindh, while in lower Sindh, patriarchal mindset is not that strong thus enabling women in lower Sindh to actively participate in decision making process at household level.

*“When a mother-in-law says that there is too much salt in the meal, then the quarrel starts. The man comes home, his mother complains that there was too much salt in meal. The husband gets up and hits his wife”*

*[FGD Khanpur]*

*“When someone kills his own wife, they go to wadhera (village chief) for protection, and in return they (husband) give their younger sister as a compensation to forgive the murder”*

*[FGD Lasbella]*

*“In joint family system, brother-in-law and father-in-law also do violence on women. Women also do violence against other women, for example, female relatives and mother-in-laws do violence against the wife of son/brother. Relatives do not respect the ‘new girl’ who enters their house after marrying their son/brother”*

*[KII Thatta]*

*“So generally patriarchal mindset is stronger in upper Sindh. Men there are so unforgiving that at times you can hear about incidents where they shoot their wives if they are seen outside their homes talking to someone, without giving her a chance to explain herself.”*

*[KII Gharro]*

### Theme II: the repercussions of VAW

According to community men and women, physical and psychological violence negatively affect family relationships by disturbing family harmony, consequently leading to distrust between couples and other family members particularly affecting women and children. The impact of VAW can further be explained into three categories, as expressed by community participants:

#### Consequences on child wellbeing

The children who suffer and witness violence at home and in surroundings are more likely to be affected mentally as compared to other community members. Moreover, the participants added that most of the children who witness violence within their families, become more aggressive as they grow, consequently become future potential perpetrators.

*“The VAW will negatively affect children’s health and the*y *start abusive language, lose confidence, become rebellious and indulge in anti-social activities.”*

*[KII Sakro]*

*“When their (men) minds are out of control due to frustration they act inhumanely with females and children, as a result, they beat their wives, mothers, sisters and children.”*

*[FGD Lasbela]*

#### Consequences on maternal wellbeing

The participants shared that VAW affects mothers leading to maternal depression which not only impacts their own well-being but also disturbs their relationships with husbands, children, close family members and other community members thereby resulting in loss of peace within the family.

*“Violence has a long term negative effect on women mental wellbeing” [FGD Peshawar]*

*“The wife is embarrassed in front of neighbors because of the inappropriate behavior and violent act of husband”*

*[FGD Peshawar]*

*“The family where violence happens, females end up facing more mental depression”*

*[FGD Mardan]*

#### Disorientation of social values

Finally, the participants added that violence within the family ultimately has a triggering effect on the whole family, community and the society, leading to disintegration of social values. Since extended family is one of the important institution in building the societal values and norms in Pakistan, if any member of the family is victimized, the whole family suffers leading to mistrust and malfunctioning within the family and society at large.

*“Violence can affect health of men and prevent peace of mind. Violence impacts all women, children, and husband” [KII Peshawar]*

*“When violence happens in a house frequently children become confused and disoriented. They become rebellious and indulge in anti-social activities like theft, substance abuse, and become a burden on society. They lose their productivity and confidence” [KII Peshawar]*

### Theme III: multiple voices regarding strategies to reduce VAW

This theme highlighted nine categories as potential strategies for reducing VAW in Pakistan and similar settings.

#### Promoting women empowerment through education

According to the participants, violence happens when a woman confronts or denies against established family and societal norms. Participants suggested that since it is woman’s primary role to bear children, therefore she should be given an equal opportunity for decisions about her life, family and children. This is only possible when women and men both are equally given awareness and education regarding attitudes and behaviors towards reducing VAW.

*“Lack of education and awareness and influence of other men’s behavior towards their wives, are among the significant barriers to combat VAW. Some men dominate over their wives and ask their wives to act like their slaves” [KII Gharo]*

*“A big change can be seen if women are given education and awareness so they can support their husbands financially and can look after children in a better way” [FGD Female, Peshawar]*

*“Education is Necessary. Miserable couples will make sure their household matters are understandable, and community education and awareness will enhance their skills which will also prepare them to cope with stressful conditions”. [KII Khanpur]*

#### Identification and recognition of women’s basic rights by the community members

Lack of awareness about women’s rights, recognition, ignorance and discouraging women at both household and community level are considered as violation of basic human rights by the community participants. Women rebelling and fighting against social patriarchy and consequently losing their lives has become more prevalent at present. Therefore, it is important that women are given respect, autonomy, recognition for their roles, and sharing of responsibilities at both household and community level. Hence it is fundamental that innovative training strategies should be initiated for both men and women for a good living environment and healthy relationships.

*“Women from upper Sindh play a very marginal role in their society, that’s why the incidents of women rebelling against the social norms and patriarchy are relatively more common there. You will hear a lot of women fighting against this openly and losing their lives as a consequence. Training programs for woman and man in regards of good living environment is of utmost importance to maintain healthy relationship among spouses.”*

*[KII Gharro]*

In fact, the community highlighted that oftentimes, women are even pulled into non-consensual/ forced sex out of the wed lock by their own husbands, which is the worst form of violence and exploitation of basic human rights. There is a dire need to create awareness with regards to women’s rights in the society.

*“I have witnessed such women, man was doing substance abuse. One of the female worker told me that a man, if don’t get drugs, they even ask other man to sleep with his wife for hundred rupees only and I have eye witness this thing. I have worked in entire district Thatta and I have seen both good and bad people and faced every type of situation. I will tell you about heroin addicts "one man was using heroine (drug) his wife told me that my husband does such things at night (similar to the above story)”*

*[KII Thatta]*

#### Improving social cohesion through team play and social relationships

It is noteworthy to mention that family members are the drivers as well as influencers of violence. Often times, family including in-laws instigate men to torture their wives. In this regard, mother-in-laws play a critical role in perpetrating VAW at household level. Therefore, the trainings and counselling to other family members for the provision of a supportive and healthy home environment can be of substantial value to reduce VAW.

Community men and women voiced that portrayal of females as weak gender, gender discrimination and inequality, patriarchal mindsets, and social issues such as early marriages are the underlying reasons that promote VAW. Discouraging these practices through advocacy channels that promote tolerance amongst men, family members and in-laws can be a potential step for reducing VAW in the society. This should also ensure that women are given the autonomy in decision making.

*“Female gender is always considered weak, but I think if you target any of them* (woman in the Family), *so it will significantly impact the community at large, normally if you do not fulfill the needs of these* (weak) *women, a positive society will not be established.”*

*[KII Peshawar]*

Family can play a substantial role in reconciliation, incases of discord among the couples. Families who take decisions with consultation and mutual understanding face fewer problems, thus reducing chances of physical and psychological violence by spouses. Relatives also play a significant role as negotiators, therefore in joint families; elders can also be included in the training sessions. A well-aware elder in the family can play his/her role as a negotiator in matters of marital conflict resolution and discouraging VAW.

*“As a woman comes to her husband’s house after marriage, she wants some rights and space for living. But as an elder person, the mother-in-law wants her rule so there is a situation of war. But in case the bride is well educated and aware and has a well-groomed personality and nature, then she will be able to manage the matters carefully”*

*[KII Khanpur]*

#### Couples’ responsibilities

The understanding of rights and tasks sharing is one of the keys for a healthy and effective relationship between husband and wife. Hence families where both men and women share responsibilities, have a more balanced life. Therefore, training and awareness for males is of utmost importance when rights of women are under consideration.

*“Men shall learn about rights of women. They shall understand that women can think as well. Sometimes I agree, and sometimes disagree. However, women have more work load and even if women work like men, they have to take care of children at the same time.”*

*[KII Peshawar]*

Lack of communication among couples living in a joint family system has the potential to lead to discord, which in turn widens differences and may result in the events of physical violence. Bridging the communication gap could possibly resolve conflicts between couples. Therefore, maintaining healthy relationships and an amicable environment at home is vastly important. Social awareness programs should be conducted and communication campaigns should be initiated to resolve conflicts through positive management.

*“To me, the first and the most important thing that needs be done is to improve communication. Communication, however, has to be positive because I’ve seen a lot of negative communication happening around us, especially due to print media (television shows and dramas).”*

*[KII Thatta]*

#### Early childhood education as a primary prevention to reduce VAW

Rearing of a child plays a critical role in shaping their attitudes and behaviors as future good or bad husbands and/or wives in the future. Therefore, education and awareness of both boys and girls from a very young age will result in good interpersonal skills development which will reduce misunderstanding and ultimately minimize VAW. Hence proper encouragement of male to treat their counterparts equally, from a young age is key strategy to nurture responsible and good man. Children who see their father beating their mother are more likely to beat their wife as well. The famous connotation; “like father like son” applies in such cases.

*“See we have learnt this in training that teacher together with parents play an important role in developing personality of a child but unfortunately, majority of parents are illiterate here.”*

*[KII Lasbella]*

#### Operationalization of community-based interventions with involvement of the local stakeholders including health care system

The community suggested that the already existing Lady Health Workers (LHWs) Program is a good medium through which community can be counseled. Since the community women are in direct contact with LHW, and maintains a good relationship with them, they can be involved in the education of females with regard to their rights and LSBE for their children. Moreover, scaling up the LHW counselling programs was also recommended by the community.

In conjunction with the training and LSBE of community through LHW, community stakeholders including religious leaders and village leaders (landlords) can also be encouraged to participate in such counselling campaigns.

*“LHWs should be assigned task to solve issues like conflict between husband and wife in which wife is more responsible”.*

*[KII Lasbella]*

*“There are number of females in contact with the LHWs as she conducts awareness sessions mostly for females, you can conduct sessions there at the Basic Health Units (BHUs). Since females come for antenatal checkups and bring their children for vaccination. Furthermore, sessions can also be arranged in Madrasa where girls are studying.”*

*[KII Peshawar]*

“*To create awareness, the role of religious leaders is important as they can highlight and preach the importance of healthy family environment to the rural community, particularly during their Friday sermons in mosques.”*

*[FGD Mardan]*

#### Revisions required in the existing laws to empower women followed by implementation

Another strategy that can have a profound impact on women’s autonomy and representation is the revisions to the existing laws and constitutions, if required. Likewise, exploring innovative avenues and adopting such opportunities for overall wellbeing of women can lead to healthy future generations.

*“There is need of revision in the existing laws dealing with issues related to women, accordingly amendments to the constitution where required As you know, in every society there are good and bad investments so we should try to explore and adopt such opportunities and empower women in our society through scholarship and job opportunities. Such investments can secure our future generations. Similarly, we have laws but lack of implementation is another reason why VAW is prevalent in the society.”*

*[KII Peshawar]*

#### Minimizing VAW through innovative approach (LSBE)

The community men and women proposed that in order to deal with the prevalent issue of VAW, there is a need to have innovative strategies which can enhance the capacity building of community men and women. Moreover, reaching out to the rural men and women for capacity building and awareness will aid the community for enhancing their understanding regarding their rights.

In this connection, the LSBE is a promising strategy to provide awareness to couples and promote healthy relationship, thus harnessing family harmony at large. For the LSBE program to have a sustainable impact, proactive publicity and promotion is required, so to widen its scope and acceptance in the community.

*“In order for women to understand their rights, there is a need for mobilization. In rural areas women don’t even know if such a thing (concept of women rights) exists. We need to reach out to these community men and women for creating awareness related to laws and legal system.”*

*[KII Thatta]*

*“We (community men and women) cannot create awareness among other fellow community members but people with sound knowledge and skills can do this through LSBE program. We can extend our support for bringing the fellow community men and women to these sessions and forum”.*

*[FGD Lasbella]*

#### Effective interpretation of religious teaching can serve as a strategy to reduce VAW

Lack of effective religious education and its misinterpretation has been shared by the community men and women as one of the underline reasons for escalating the issue of VAW. Further, they emphasized that the acts of VAW can be minimize with the effective practice of religious education as misinterpretation of religious teachings has led to false beliefs considering: women are inferiors, which is in contrary to the religious teachings. The Islamic teachings have highlighted the role of women and considers both men and women equal and effective members in a society.

*“Islam clearly mentions the rights of a woman, as mentioned in the Holy Quran. Men and women should follow teachings of Islam. Our Holy Prophet (PBUH) has always valued the specific position Islam gives to a woman in the society. A woman can be a mother, daughter or sister.”*

*[KII Rahimyarkhan]*

*“The people interpret religion in a wrong way as Islam has never regarded women as inferior. If it was so, then our Holy Prophet (PBUH) would not have valued the contribution of his wives and daughter. The people do not give confidence to their women and daughters to solve their issues through negotiations. They prefer and force their women to stay at home and believe it is better to suppress women”.*

*[KII Lasbella]*

## Discussion

This study is first of its kind that has documented the perceptions and attitudes of community stakeholders with regards to VAW in Pakistan, to be able to suggest what cultural interventions are feasible to prevent and detect the VAW. Largely, the result of this study is adherent to the literature that women are the victims of violence and men are the most cited perpetrators. However, mother-in-laws and other relatives also perpetrate VAW. Community members and stakeholders’ views were consistent with the evidence. In addition, the study findings also strengthened the context-specific understanding of VAW in Pakistani context using ecological model.

### Contextual perspectives of factors, types, reporting and consequences of VAW

Our study findings reiterated that women are victim not only by male members of the society, but also by other immediate family members and their in-laws. Majority of the community members’ understanding of VAW wasn’t limited to physical torture only. In fact, the community members consider psychological violence as a distinct form of Violence. A study conducted in Hyderabad highlighted that around 47% of the women in Pakistan report some form of abuse by their husbands such as physical, verbal or sexual abuse. However, only 21% of the women reported controlling behaviors, which is also the form of VAW, whereas 20% of the women reported any conflict with in-laws, as another form of VAW [20]. Our study findings are consistent with previous research in detecting that a large proportion of women acknowledged being subjected to violence at some point through the course of their lives with various types of abuse. Despite of having knowledge about existence of different forms of violence, the community however, refrains from reporting these ill-treatments. According to a study conducted showing that women who are subjected to violence, 64% of these women remained silent against verbal abuse, while 48 and 73% women remained silent for physical abuse and sexual abuse respectively. Same study further highlights that only 4% of the women returned to their parents ‘home after verbal abuse, however, 10% of the women returned to their parents after physical abuse [21]. The present study highlighted that the most common reason for remaining silent includes the fear of violence being intensified upon reporting. Other reasons for remaining quiet against violence were lack of hope and inter-family marriages i.e., having their siblings married in the same family. Many women consider it as a private matter and do not report it to others due to honor/respect [20].

However, in our country there is the existence of Domestic Violence Act 2012, and explicitly criminalizes any act of VAW distinctively covering physical, psychological as well as verbal abuse, its lack of implementation has also resulted in the unfamiliarity within the community [21]. This results in low registration of the cases of domestic violence in Pakistan representing ice burg phenomena. Underreporting of such acts is driven by patriarchy and feudalism where women’s own families dissuade speaking against maltreatments by the husbands and their families. From the ecological Model’s perspective, since the application of law is insufficient, this has significant triggering effect at women/individual level. These mindsets are common throughout the region and beyond as revealed by a study on the subject amongst women living in the United Kingdom, where lack of support for women was the major influence underlying their decision to stay in violent marriages. Women stated that they are coerced to embrace violence and hide the persecution to avoid shame and fear of other community members finding out [21].

Stakeholders and community members also shared that VAW is an interaction of several constructs as per explained by Ecological model that it is ranging from societal factors like men dominancy, accepting violence as a way to resolve conflict; at Community Level like poverty, low socio economic status; at relationship level like marital conflicts, decision-making in the family and at Individual level like being female, unemployed or being the house wife. This represses gender roles and enhances gender Gap with in the Pakistani society at large [22].

Present study also reveals the interesting yet highly neglected site of VAW in this region that is the failure of the society to recognize the impacts of violence on women’s mental health. The prevalence of mental illnesses among those women who experience violence is notably high, yet little attention has been consecrated to the recognition and mitigation of the impacts of violence on the physical, mental and social well-being of the victims. These findings are adherent with the evidence from a qualitative study that explored consequences of spousal abuse on women’s psychological health, study states that women verbalized behavioral reactions such as crying spells, hopelessness, irritability and impatience. Besides these psychological symptoms, women experienced low self-esteem by verbalizing shame, guilt, self-pity, feeling of insecurity and powerlessness. They developed negative perception about all men in the world due to their spouse’s attitude towards them. They also had psychosomatic reactions and physical symptoms [23]. Our study also validates that these psychosocial and mental manifestations disrupts the social structure of the communities and the society alongside putting the health and well-being of the individuals and their relationships in jeopardy.

### Stakeholder’s recommendations to identify strategies to reduce VAW

At the wider societal level, our study indicates an urgent need to align the existing interventions and programs with the emerging needs of the society with regards to reduction to violence. One of the common strategy denied by the community is that the existing healthcare system in Pakistan should embraces community-based interventions to reduce VAW such as through involvement of frontline community health workers (Lady Health Workers Program or Community Midwifery program) who provide primary health and counselling services on maternal and child health, immunization, nutrition, WASH, etc. One such intervention was tested in peri-urban areas of Karachi where these lady health workers were mandated to deliver “Family life education sessions” in the community, particularly, to women, men and adolescents. The midline evaluation report of the program unveiled substantial improvements in community’s knowledge pertinent with maternal health and rights [24]. It is therefore recommended that such initiatives should be scaled up and operationalized whereby training and counselling of families to provide a supportive environment is endorsed, as it can be of substantial value to reduce VAW. Since family can play a substantial role in reconciliation, consultation and mutual understanding among households [25], thus these trainings could be favorable in reducing physical and psychological violence by spouses. Our study also emphasized the need for integration of pragmatic strategies to combat VAW through existing education system. For instance, integration of gender equality in early childhood education could be highly beneficial in promoting sustainable change in the society. A qualitative study from Pakistan recapitulated the importance of gender identities in shaping the behaviors of individuals in their latter lives thereby indicating towards the need for instilling positive gender identities from early childhood. Our study suggest that the educational interventions must involve teachers in these training programs as well since the perceived norms and subjective experiences of these teachers have a notable influence on their preaching. Further, the variation in expectation of the teachers from the two genders also strengthen these negative gender norms [26]. A cross-country analysis of early childhood textbooks shows that early childhood gender identities are further strengthened when children read their textbooks in which there are specific characteristics and jobs assigned to each gender. These roles create gender stereotypes and strengthen conservative gender norms in children’s minds [27]. Though stereotypical behaviors and specific expectations from each gender are common throughout the world. In Pakistan, it has been observed that male members are allowed to enjoy more rights than women and that starts from a very young age even in the feeding practices. In this regard, our study recommends a dire need for revamping of primary schools’ infrastructure and the curriculum. Likewise, training of the parents to practice more gender neutral practices in the upbringing of children would be a significant intervention to promote gender equality as stated by stakeholders in this study.

A secondary analysis of Pakistan Demographic and Health Survey 2012–13 that investigated association of empowerment with VAW revealed that adherence to conservative gender norms, lack of economic decision-making power, and lack of higher education were found strongly associated with VAW in the past 12 months. Sticking to traditional conservative gender norms among women was also significantly associated with experiencing emotional violence as well as controlling behaviors by husbands [28]. Therefore, it is essential to raise awareness among women regarding liberal gender norms and educating women about their rights and safety. This can be effectively done by providing life skills education trainings to the women as well as men as stated by community stakeholders. Evidence suggests community and group interventions involving women and men can shift discriminatory social norms to reduce the risk of violence [1]. There must be multi-pronged interventions targeting holistic empowerment through economic as well as social skills building. These programs should focus on revisiting gender roles and expectations. In addition, there is a strong need of support for survivors. This support should be available for legal actions as well as seeking help for information of various types of actions available during the course of conflict.

Moreover, when women do not seek help and are accustomed to remain silent, the majority of them are unable to decide when to seek official help from either police or law enforcement agencies. Mostly, police is informed by the neighbors or relatives when the situations are as extreme as it could affect life of the women or children [29]. Furthermore, another area identified in the study as a key strategy was to effectively interpret the religious teachings to reduce VAW. Evidence from studies conducted in other countries also identified that religious misinterpretations alongside cultural norms and the ineffective implementation of laws have led to a weak status of women therefore it is challenging for women to speak for their opinions and rights. Religious attitude therefore, had a significant relationship with the type and duration of VAW [30–32]. Lastly, it is suggested that a multisectoral approach must be used to sensitize the community on not only the reporting of violence but also on reevaluating the societal norms that support persecution of women.

### Linking of VAW with ecological model and PRA

This study used the Heise model to explain the factors that promote interpersonal violence at the individual, as well as the societal level. Our study drew attention on the interfacing of the individual, the relationship, the community, and the societal constructs in promoting violence and shaping behaviors of the perpetrators and the victims in response to violence. Community participation at each level could help us in identification of strategies that could be utilized to minimize VAW.

Our study proposes that individual interactions at schools, workplaces, neighborhoods and even with the healthcare providers can promote sustainable positive impacts on the violent behaviors, as the feedback collected from all community stakeholders shared their experiences in relations to these constructs. In addition, analyzing the understanding of community regarding impact of VAW there is high need of raising awareness of VAW, existing laws and basic life skill education at every level.

### Strengths and limitations

This study was carried out across the four provinces of Pakistan which helped in gaining in-depth understanding of the concept of VAW. Therefore, this information will help to formulate culturally appropriate and acceptable interventions across Pakistan. This study utilized the Ecological Model and Participatory Research Action (PRA) concomitantly to explore the multifaceted phenomenon of violence at all levels of embodiment thereby enabling the researchers to unearth the interrelation of several construct at the four level, while involving all relevant stakeholders in the identification process.

The use of PRA approach also allowed the researchers to make qualitative enquiries to gain an in-depth understanding of existing status of the women in the family and the impact of this status over her life across various stages of development. As such, the interaction with multiple stakeholders also facilitated the identification and clustering of multiple intervention startegies at each ecological level and the reasons why a few existing interventions failed to adequately address VAW in Pakistan. Consequently, this enhanced the learning process and may assist the progress of locally designed interventions that are culturally relevant and saleable.

## Conclusion

Women are exposed to violence in all the provinces of Pakistan. A list of community-based interventions has been suggested to combat VAW from the eyes of community stakeholders. However, the responsibility to bring about change in behavior and attitudes must begin with engaging men in all the interventions that aim to reduce violence. There is a dire need to implement the interventions that are locally planned, to captured local cultural factors contributing to VAW and scalable within the existing system in Pakistan.

## Data Availability

The datasets used and/or analyzed during the current study are available from the corresponding author on reasonable request. In order to maintain confidentiality, the names and information of the participants will be kept anonymous and will be given a unique identifier.

## Abbreviations

AKU: Aga Khan University
BBCs: BISP Beneficiary committees
BISP: Benazir income support program
CT: Cash transfer
COE: Center of Excellence Women and Child Health
DALYs: Disability adjusted life years
FGDs: Focus group discussions
KIIs: Key informant interviews
KPK: Khyber Pukhtunkhuwa
LHWs: Lady Health Workers
LSBE: Life skills based education
NGOs: Non-Governmental Organizations
PRA: Participatory research action
QALYs: Quality adjusted life years
SONAM: School of Nursing and Midwifery
SDGs: Sustainable development goals
VAW: Violence against Women
WASH: Water Sanitation and Hygiene
